# Structural and single-molecule insights into the core human mitochondrial DNA replisome

**DOI:** 10.1042/BCJ20260373

**Published:** 2026-08-12

**Authors:** Ismael Plaza-G.A., Samuel Miguez-Amil, Allison M. Hayes, Grzegorz L. Ciesielski, Rafael Fernandez-Leiro, Borja Ibarra

**Affiliations:** 1 Instituto Madrileño de Estudios Avanzados en Nanociencia, IMDEA Nanociencia, Madrid, Spain; 2 Structural Biology Programme, Spanish National Cancer Research Centre (CNIO), Madrid, Spain; 3 Department of Biology, University of North Florida, Jacksonville, FL, U.S.A.; 4 Nanobiotecnología (IMDEA-Nanociencia), Unidad Asociada al Centro Nacional de Biotecnología (CSIC), Madrid, Spain

**Keywords:** DNA replication, mitochondria, protein structure, single-molecule

## Abstract

Replication of human mitochondrial DNA (mtDNA) is essential for the maintenance of oxidative phosphorylation and cellular energy homeostasis. Impairment of this process leads to mtDNA deletions, depletion, and point mutations that underlie a broad spectrum of mitochondrial diseases, as well as contributing to neurodegeneration, aging, and cancer. The core human mitochondrial replisome, composed of DNA polymerase γ (Polγ), the replicative helicase Twinkle, and the mitochondrial single-stranded DNA-binding protein (mtSSB), is the main complex responsible for replicating the mitochondrial genome through a highly coordinated yet still incompletely understood mechanism. Mutations in the nuclear genes encoding these proteins represent the most common cause of inherited disorders affecting mtDNA maintenance, underscoring the importance of understanding their coordinated molecular function. Recent advances in cryo-electron microscopy and single-molecule approaches have provided unprecedented insight into the structural organization and dynamic operation of the core components of the mitochondrial replisome. These complementary methods are establishing a quantitative mechanistic framework for understanding how the mitochondrial replisome initiates, progresses, and regulates the replication of the light and heavy strands of mtDNA. In the present review, we integrate recent structural and single-molecule findings to describe the mechanisms governing the activity of Polγ, Twinkle, and mtSSB at the mitochondrial replication fork, and discuss remaining challenges toward reconstructing a complete mechanistic model of human mtDNA replication.

## Introduction

Mitochondria depend on the faithful maintenance and expression of mitochondrial DNA (mtDNA) to sustain oxidative phosphorylation and cellular energy homeostasis. The mitochondrial genome encodes polypeptides essential for the OXPHOS system [1], and defects in mtDNA maintenance result in energy deprivation and may lead to the development of human disorders. Depending on the extent of damage, mtDNA-associated diseases range from localized neuromuscular disorders, such as progressive external ophthalmoplegia, to more aggressive multisystem disorders, such as debilitating sensorineural hearing loss with ovarian insufficiency in Perrault’s syndrome or early-onset encephalopathy with prominent seizures, liver involvement and short life expectancy in Alper’s syndrome [2–4]. Defects in mtDNA have also been implicated in other, more prominent, disorders such as Parkinson’s, Alzheimer’s, and other neurodegenerative diseases, or various types of cancer [5–7]. While investigations are ongoing [8], there is currently no available cure or effective treatment for any mitochondrial DNA-associated disorders [6]. Despite the relatively high prevalence of pathogenic mtDNA variants in the population (i.e., 1:200) [9], clinical disease manifests less frequently (∼1 in 2000), reflecting the substantial tolerance of mitochondria to partial genome dysfunction [10].

**Figure 1:**
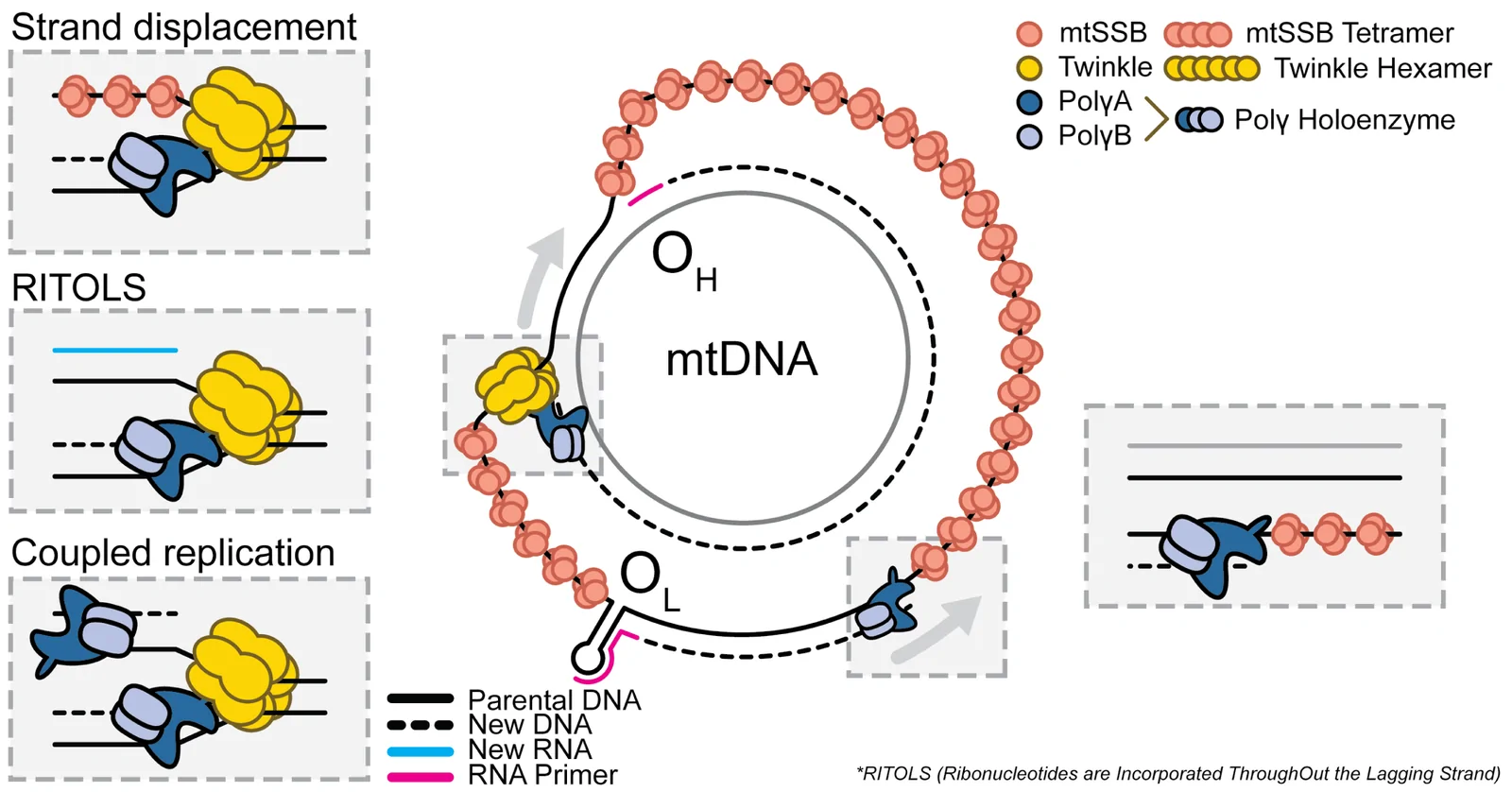
Strand displacement model of mtDNA replication Replication of mtDNA is initiated at the heavy strand origin of replication (O_H_) by mitochondrial RNA polymerase. The resulting RNA primer is then transferred to the Polγ holoenzyme, Twinkle, and mtSSB. H-strand replication subsequently proceeds unidirectionally. When the replisome passes the light strand origin of replication (O_L_), a stem-loop structure forms and is recognized by mitochondrial RNA polymerase, which primes synthesis of the mtSSB-coated light strand. Light-strand synthesis is carried out by Polγ. The left panels show the putative organization of the core replisome factors at the fork according to alternative replication models proposed in the field.

The relevance of mtDNA replication is underscored by the fact that the most common causes of mitochondrial pathologies are mutations in the nucleus-encoded core components of the human mtDNA replisome [11]. This includes DNA polymerase γ (Polγ), the replicative helicase Twinkle, and the mitochondrial single-stranded DNA-binding protein (mtSSB) [12] (Figure 1). Pathogenic variants in POLG (Polγ), C10orf2 (Twinkle helicase), and SSBP1 (mtSSB) impair enzymatic activity, reduce processivity, or destabilize their cooperation during DNA synthesis, ultimately causing mtDNA deletions or depletions [13–16]. Although biochemical studies have provided major insights into the functional consequences of replisome mutations, the lack of structural and mechanistic detail needed to explain pathogenicity in molecular terms has hindered the development of effective therapeutic strategies.

Human mtDNA is a ∼16.6 kb-long circular genome, with the characteristic ∼650 nt-long displacement loop (D-loop) present in actively replicating molecules [17]. The replication process proceeds predominantly via the asymmetric strand-displacement model (Figure 1) [18–20]. It originates from a single origin and advances unidirectionally, producing the leading nascent strand. The fully assembled replisome is essential for the DNA fork advancement in this first phase, as the insufficient presence of Twinkle helicase appears to limit synthesis of the leading strand beyond the D-loop [21].

With the advancement of the replication fork, the parental non-template (i.e., heavy) strand remains displaced, and concurrent explanations have been proposed for the way it may be stabilized (Figure 1). In the Clayton variation of the strand displacement model [22], the displaced strand is progressively coated by mtSSB and, more recently, this scenario has been supported by the strand-specific ChIP-Seq analysis of mtDNA occupancy [19]. In the alternative variation by Holt et al., the displaced strand is progressively coated by stretches of RNA (i.e., ribonucleotides are incorporated throughout the lagging strand, RITOLS), which are sourced from pre-existing transcripts [23]. This model stems from the analysis of mtDNA replication intermediates by 2D agarose gel electrophoresis and the observation of prominent double-stranded fork DNA species (i.e., the Y-arc), which are RNase H-sensitive [24]. According to this model, RNA is being incorporated onto the displaced strand during replication via the bootlace mechanism [20] and although enzymatic aspects of the process have not been established so far, it has recently been demonstrated that Twinkle helicase preferentially anneals RNA onto the DNA fork [25]. Interestingly, mtSSB appears to inhibit the capacity of Twinkle to anneal RNA [25], which opens the possibility that the two variations can coexist and be reciprocally regulated. After replication has progressed through roughly two-thirds of the mtDNA circumference, the origin of light-strand replication is exposed, and the displaced heavy strand forms a stem-loop structure that serves as the origin for light-strand replication (Figure 1) [26]. The precise mechanism of DNA synthesis over the displaced strand remains less understood. While it likely occurs without participation of the Twinkle helicase, other auxiliary factors may assist Polγ, eliminating secondary structures, RNA hybrids, and other replication barriers on the displaced template strand. It appears that lagging-strand synthesis is a particularly vulnerable stage of the process, prone to aberrations that lead to strand breakage and mtDNA deletions [27–29]. While the formation of deletions can be reconstituted *in vitro* [30], the circumstances underlying this process *in vivo* remain unknown [31]. Although alternative models (e.g., strand-coupled [32]) have been proposed (Figure 1), the body of biochemical evidence [33], and especially the recent direct visualization of individual replicating mtDNA molecules (mito-SMARD [34]), provided unequivocal evidence for the asymmetric asynchronous replication. Whether mtSSB or RNA coats the displaced strand remains to be further investigated.

Recent advances in cryo-electron microscopy (cryo-EM) and single-molecule approaches now provide complementary frameworks for understanding the molecular processes that govern the activity of the core replisome factors and the rationalization of pathogenic mechanisms at high resolution. While cryo-EM reveals near-atomic structural organization, single-molecule techniques allow direct observation of dynamic biochemical processes, bridging genotypes to biochemical phenotypes [35] and raising hope for accelerated development of targeted therapeutics. Indeed, the recent breakthrough development of the first Polγ stabilizer (PZL-A) was supported by structural analysis of the drug–Polγ complex [8]. In the following sections, we integrate recent structural, biochemical, and single-molecule studies to provide a mechanistic framework for understanding the coordinated operation of the core human mitochondrial replisome factors and how their dysfunction leads to mitochondrial disease.

### mtSSB

The mtSSB, encoded by SSBP1, is essential for mitochondrial DNA replication. Genetic disruption of SSBP1 or its homologs results in severe mtDNA depletion and respiratory deficiency [16]. Knockdown and conditional knockout experiments in human cells and animal models demonstrate that mtSSB is essential for viability, with pathogenic variants compromising its ability to coordinate the activity of Polγ and Twinkle [36]. mtSSB also plays a role in stabilizing single-stranded DNA (ssDNA) intermediates during replication [19] and primer formation [36], as well as in the maintenance of the D-loop structure [37].

Binding of mtSSB to ssDNA enhances the activity of its replication partners Polγ and Twinkle through several mechanisms, including decreasing template secondary structure formation, reducing replication-fork regression pressure, facilitating fork recognition, and minimizing inhibitory ssDNA contacts [36–43].

**Figure 2:**
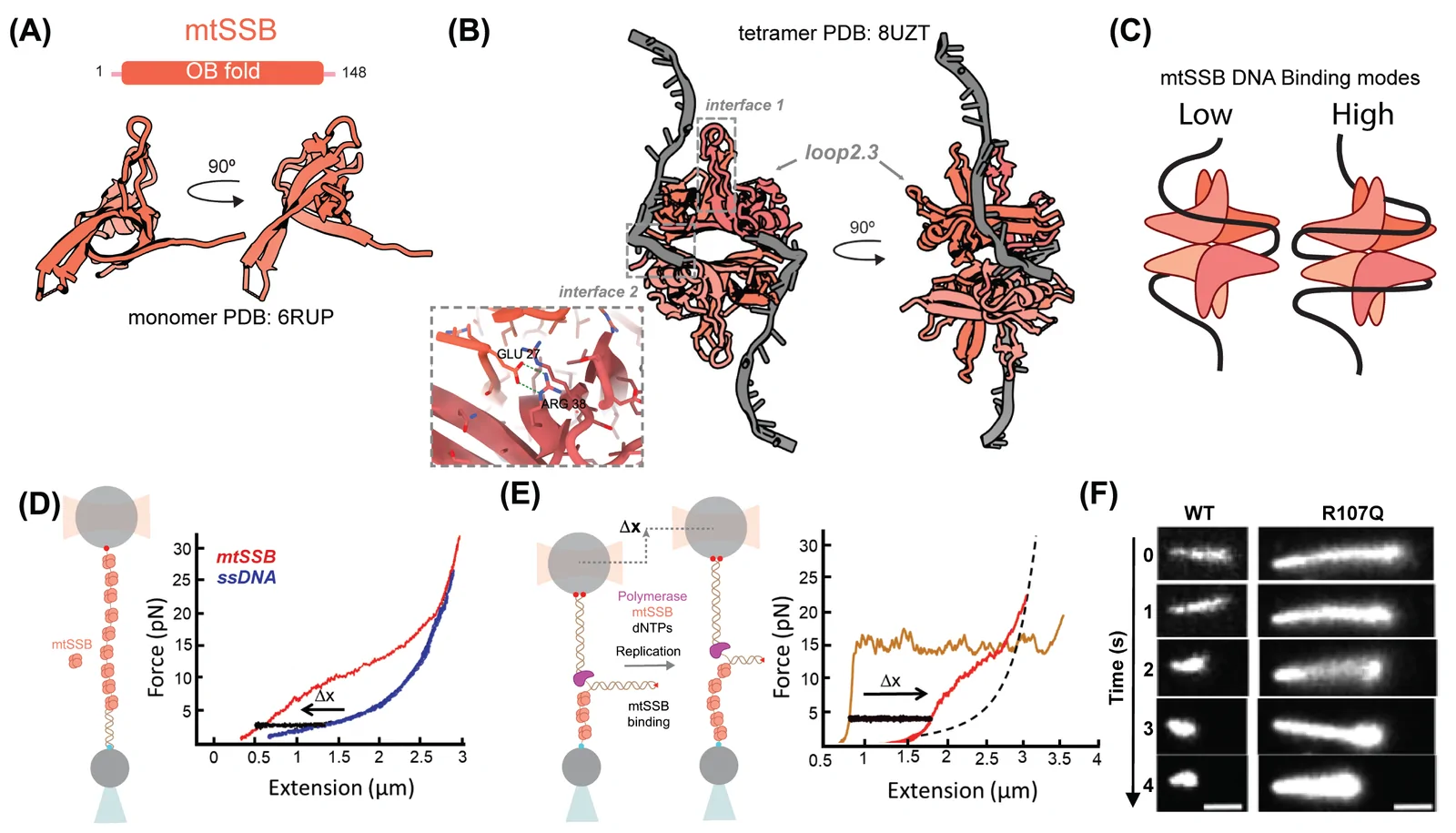
mtSSB structure and binding modes (**A**) Domain schematic representation of mtSSB (amino acids 1–148) highlighting the OB-fold domain (amino acids 26–141) (PDB: 6RUP) [49]. (**B**) Cartoon representation of the mtSSB tetramer bound to two ssDNA oligonucleotides (PDB: 8UZT) [44]. The positions of loop 2.3 are highlighted. The box shows residues at the tetramerization interface. (**C**) Schematic diagram showing ssDNA wrapped around the mtSSB in the *low*- (left) and *high*-binding (right) modes. (**D**) (*Left*) Schematic of the optical tweezers experimental setup to manipulate individual mtSSB–ssDNA complexes. (*Right*) Force-extension curves of an ssDNA molecule (5080 nt) in the absence (blue) and presence (red) of mtSSB (50 nM). At constant force (∼3 pN), wrapping of ssDNA by mtSSB results in a decrease in the distance between the beads (Δx, black). (**E**) (*Left*) Experimental configuration used to monitor co-replicational binding of mtSSB to ssDNA using optical tweezers. Strand displacement DNA synthesis by Phi29 DNA polymerase was initiated at a constant force (3 pN) in the presence of mtSSB. As replication proceeds, the displaced ssDNA strand becomes gradually coated by mtSSB. Strand displacement synthesis terminates at the abasic site (red triangle), producing an ssDNA segment bound by the mtSSB. (*Right*) Force-extension curves of the initial DNA hairpin tether (brown) and the final DNA molecule gradually coated by mtSSB. The extension change associated with replication and mtSSB binding (Δx) is shown in black. In panels D) and E), fits of the final force-extension curves using the appropriate polymer elasticity models were used to determine the mtSSB binding mode on ssDNA. Adapted from [41]. (**F**) R107Q mutation alters the wrapping geometry of mtSSB on ssDNA. ssDNA molecules pre-incubated with fluorescence-labeled mtSSB^Alexa555^ (WT or R107Q) were stretched by buffer flow to a nearly constant length (Time 1). Addition of 100 mM NaCl progressively shortened the protein–DNA complexes to different extents (Times 2–4). Quantitative analysis of the apparent contour length together with the total fluorescence intensity of individual molecules showed that mtSSB^WT^ induces stronger ssDNA compaction than mtSSB^R107Q^. Adapted from Martucci et al. [50].

Structurally, mtSSB is a 148-amino acid protein that readily forms homotetramers, with each monomer having a molecular weight of 16 kDa [44–46]. The quaternary structure is organized as a dimer of dimers: each dimer is stabilized by two intermolecular antiparallel β-strands, while the tetramer is maintained by four intermolecular salt bridges between dimers [47,48] (Figure 2A). In addition, a divalent cation at the dimer-dimer interface has been proposed to contribute to tetramer stability (PDB: 6RUP) [44].

mtSSB is homologous to *Escherichia coli* SSB (*Eco*SSB), and both proteins share a conserved oligonucleotide/oligosaccharide-binding (OB) fold that forms a high-affinity ssDNA-binding pocket in each monomer [44]. However, unlike *Eco*SSB, mtSSB lacks the acidic, intrinsically disordered C-terminal tail that mediates protein–protein interactions and contributes to negative intratetramer cooperativity during ssDNA binding in *Eco*SSB [51]. This negative cooperativity limits the simultaneous binding of two short ssDNA molecules within the same *Eco*SSB tetramer. In addition, mtSSB contains a flexible loop (loop 2.3, Figure 2B) only found in vertebrates [52]. This loop mediates functional interactions with Polγ, facilitating the release of the mtSSB protein from the DNA during replication of the light strand, as described by single-molecule manipulation experiments with optical tweezers [38].

Recent X-ray crystallography studies identified the key residues within the OB fold binding grooves that guide ssDNA through electrostatic and hydrophobic contacts [44]. These studies showed that ssDNA wraps around two asymmetric regions, which reside on different monomers within the same mtSSB tetramer, but not in the same dimer (Figure 2B). The position of these DNA-binding regions on adjacent protomers enables the ssDNA to traverse the interface between subunits, facilitating high affinity and structural stability. Together, these regions bind 17 nt very tightly, with additional, less tight contacts extending the bound length to ∼30 nt.

Analysis of the force-extension curves of individual mtSSB-ssDNA complexes obtained with optical tweezers showed that mtSSB organizes ssDNA in at least two different binding modes (Figure 2C). High protein concentrations and/or low-salt conditions promote the *low*-binding mode that wraps ∼40 nt per tetramer, consistent with crystallographic findings, whereas the *high* mode wraps ∼60 nt per tetramer and dominates at low protein concentrations and/or high-salt conditions [41]. These two different binding modes were further corroborated by bulk biochemical studies [51] and resemble those previously described for *Eco*SSB [53].

The different binding modes leave varying amounts of DNA accessible to other proteins, suggesting that they may have distinct functional roles. In line with this hypothesis, single-molecule fluorescence microscopy and acoustic force spectroscopy showed that disease-associated R107Q mutation alters the DNA-wrapping and compaction properties of mtSSB [50]. In addition, optical tweezers studies demonstrated that the low-binding mode of mtSSB is favored over the high-binding mode during DNA replication [41] (Figure 2D,E). Together, these findings support the idea that the distinct mtSSB binding modes fulfill specialized functions, as previously proposed for *Eco*SSB [53,54,55].

### DNA polymerase γ

**Figure 3:**
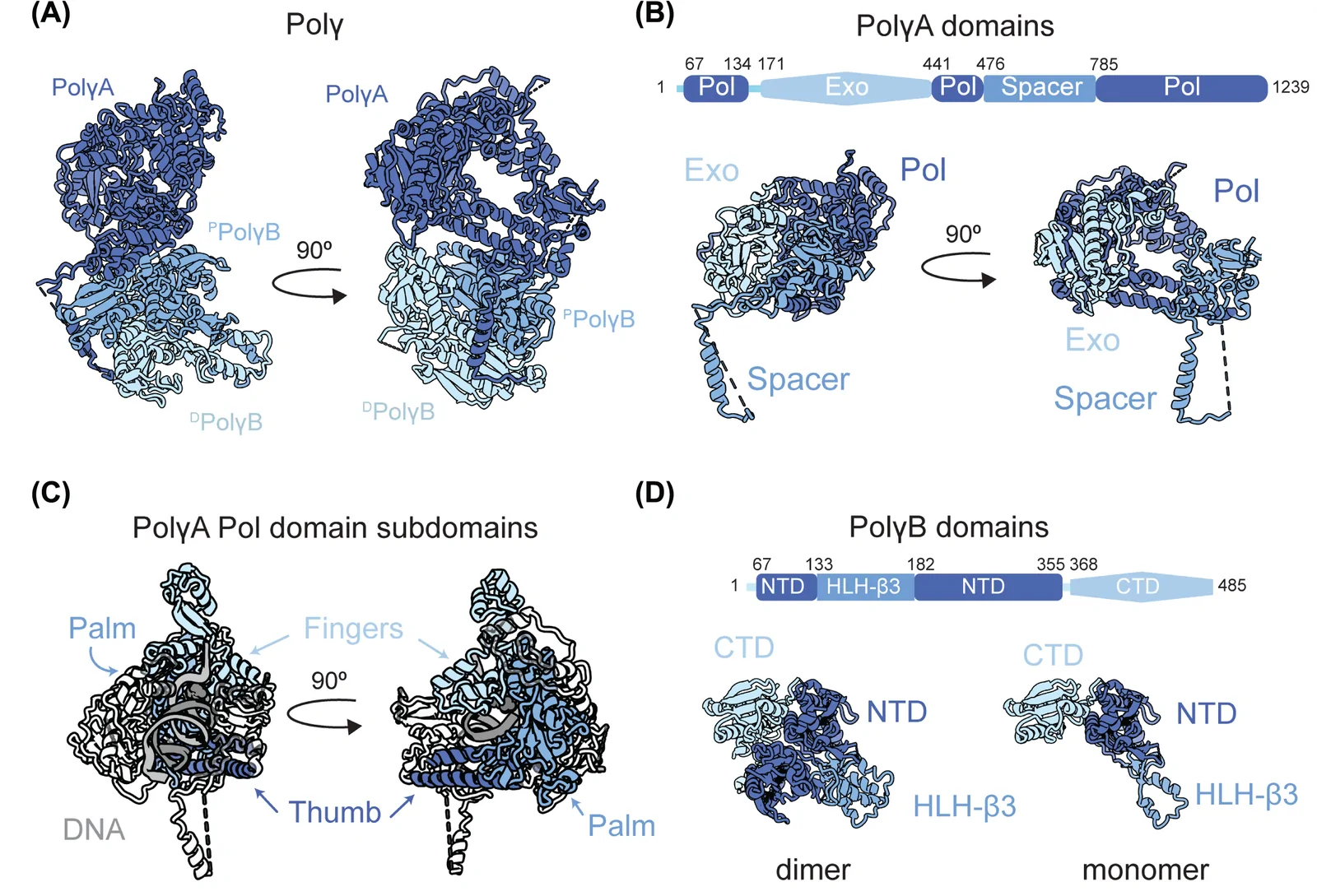
Polγ structure and domain organization (**A**) Overall structure of the Polγ holoenzyme, where the monomeric PolγA and the dimeric PolγB are represented (PDB: 8D33) [61]. (**B**) Schematic representation of the domains of the Polγ catalytic subunit. The polymerase (Pol), exonuclease (Exo), and the Spacer domains are indicated in both the schematic and structural representations. (**C**) Functional subdomains of the Polγ polymerase domain, which adopts the classical right-hand fold with thumb, palm, and fingers subdomains. (**D**) Schematic representation of the PolγB subunit. The N-terminal domain (NTD), C-terminal domain (CTD), and helix–loop–helix β3 dimerization (HLH-β3) domain are indicated in both the schematic and structural representations of the dimer and monomer.

Polγ is the only replicative DNA polymerase found within the mitochondria. The holoenzyme is composed of two subunits (Figure 3A): a monomer of the catalytic subunit PolγA encoded by the gene POLG [56] and a homodimer of the accessory subunit, PolγB [57], encoded by the gene POLG2 [58]. Pathogenic variants in both the catalytic and accessory subunits of Polγ have been identified in numerous mitochondrial disorders, such as progressive external ophthalmoplegia (PEO), Alpers syndrome, and other mtDNA depletion syndromes [5,59]. Furthermore, impaired proofreading activity of the catalytic subunit PolγA has been shown to cause premature aging phenotypes in mammalian models [60].

PolγA is a multifunctional protein, combining three catalytic activities: 5′ to 3′ DNA polymerase for primer extension, 3′ to 5′ exonuclease for proofreading, and 5′-deoxyribose phosphate lyase for base excision repair [62]. Structurally, PolγA is divided into three domains (Figure 3B). These include a polymerase domain, which adopts the canonical ‘right-hand’ fold with palm, fingers, and bipartite thumb subdomains, and the exonuclease domain, which excises misincorporated nucleotides to maintain fidelity. The third domain is the spacer domain, which contains both accessory-interacting determinant (AID) and intrinsic processivity subdomains. The latter is responsible for the interaction with PolγB, while the former provides additional contact points to double-stranded DNA (dsDNA) [63] (Figure 3B).

PolγB, though catalytically inactive, is indispensable for the processivity of PolγA, increasing the efficiency of nucleotide incorporation by nearly 100-fold [64,65]. The two monomers of PolγB are referred to as proximal (^P^PolγB) and distal (^D^PolγB) PolγB, depending on their structural proximity to PolγA [66] (Figure 3A). Early studies elucidated the protein–protein interaction interfaces and domain architecture of both PolγA and PolγB [57,63,67,68]. PolγB can be topologically divided into NTD, which includes an HLH-β3 domain, and CTD (Figure 3D). Notably, the interaction between PolγA and PolγB is asymmetrical across the two subunits of PolγB, with PolγA primarily interacting with the ^P^PolγB through the thumb and AID domains, while forming only two salt bridges with the ^D^PolγB [63]. Although the accessory subunit PolγB has been shown to be capable of binding dsDNA independently of PolγA *in vitro* (44 ± 7.3 nM) [69,70], its major role is to enhance and modulate the DNA binding and activities of PolγA [71], while its ability to bind dsDNA independently may also influence the organization of the polymerase–DNA complex.

The Polγ holoenzyme exhibits the canonical primer-dependent DNA synthesis activity of family A polymerases and a significant strand displacement activity, which is relevant for primer removal [72]. In addition, the catalytic subunit, PolγA, has 3′–5′ exonuclease [73] and phosphate lyase activities [62]. All these activities make Polγ a multifunctional enzyme capable of DNA replication, proofreading [74], base excision repair of apurinic/apyrimidinic sites [75], clearing of G-quadruplexes in the template strand [76], and degradation of aberrant mtDNA molecules [77,78]. Unlike the multi-subunit nuclear replicative polymerases Polδ and Polε, Polγ must balance all these capacities within a single catalytic core, necessitating exceptional versatility and conformational plasticity. Although the precise mechanisms by which this single protein complex coordinates such a diverse array of functions remain to be fully elucidated, advances in structural biology, biochemistry, and single-molecule analysis have provided important mechanistic insights into its multifunctional roles.

#### DNA synthesis: primer extension

The polymerase domain synthesizes DNA by adding complementary nucleotides to the 3′ end of the primer strand, following the canonical mechanism for DNA polymerases. Multiple crystallographic and cryo-EM structures have shown that the formation of the new phosphodiester bond is mediated by two Mg^2+^ ions that facilitate nucleophilic attack on the incoming nucleotide and stabilize its triphosphate moiety within the palm and fingers subdomains [67,68], involving residues D890, R943, K947, and D1135.

**Figure 4:**
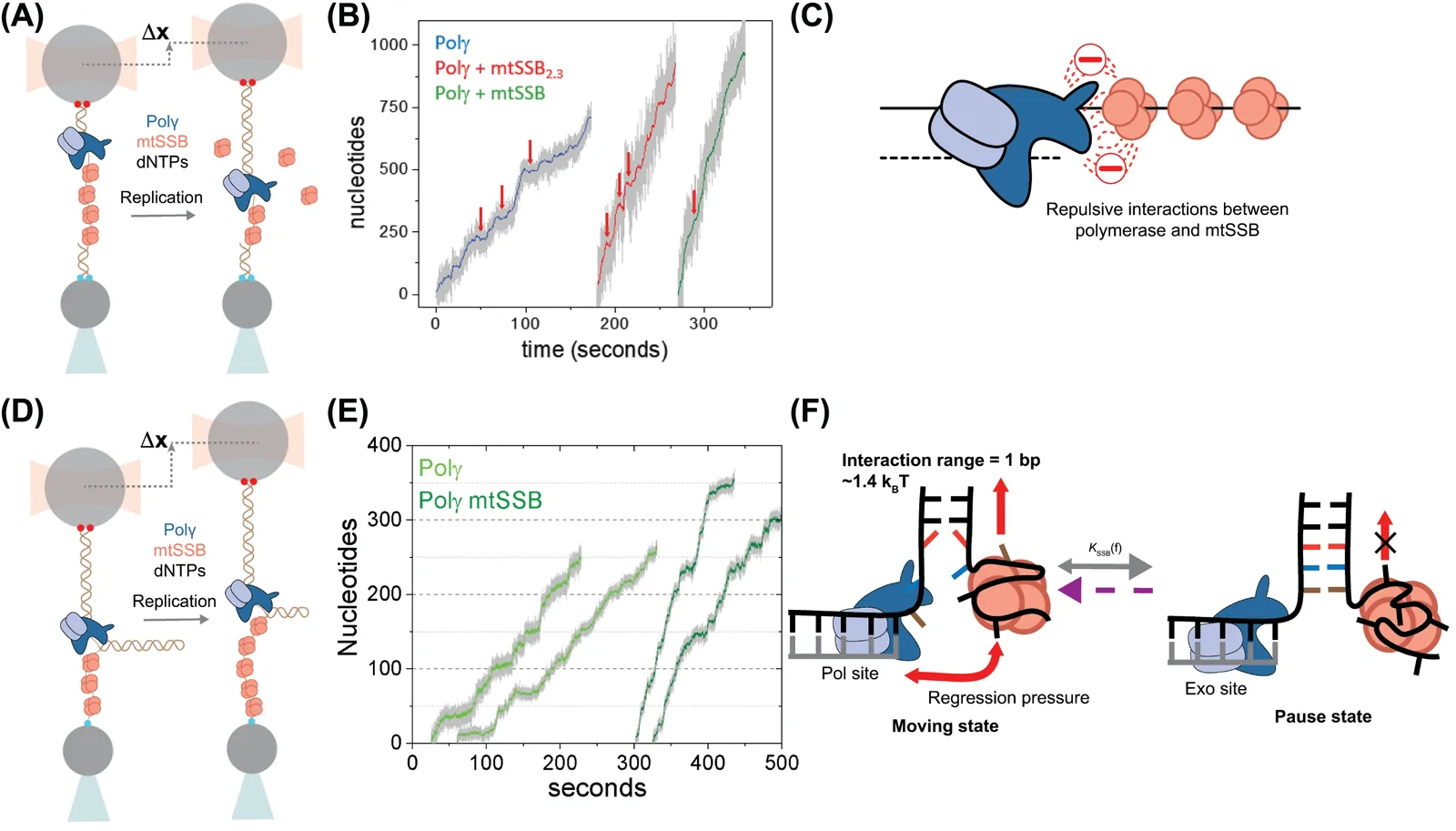
Single-molecule measurements of Polγ primer extension and strand displacement activities (**A**) Optical tweezers experimental setup to measure Polγ primer extension activity in the presence of mtSSB. At constant force, the activity of Polγ on a primed DNA molecule tethered between a bead in the optical trap (light red cone) and a bead on top of a micropipette (light blue) changes the end-to-end distance of the tether (Δx). This activity can be measured in real-time with nanometre and millisecond resolutions. (**B**) Representative replication traces of Polγ on mtSSB-free ssDNA (blue) and ssDNA covered either by wild-type (green) or mutant mtSSB (red). Individual traces show periods of activity separated by transient inactive pause events (red arrows). (**C**) Analysis of the differential effects of wild-type and mutant mtSSB on the real-time replication kinetics of Polγ suggested that electrostatic repulsion facilitates the removal of mtSSB by Polγ during light-strand replication (adapted from [38]). (**D**) Optical tweezers experimental setup to measure Polγ strand-displacement activity on a single DNA hairpin tethered between a bead in the optical trap (light red cone) and a bead on top of a micropipette (light blue). At constant force, strand displacement DNA synthesis by Polγ increases the end-to-end extension of the hairpin (Δx). (**E**) Representative strand-displacement traces of Polγ in the absence (light green) and presence of mtSSB (dark green). (**F**) Diagram illustrating a two-state model in which Polγ alternates between an active polymerization or moving state (left) and an inactive or paused state (right). In the moving state, mtSSB decreases the fork regression kinetics and the holoenzyme–mtSSB complex destabilizes the first base pair of the DNA hairpin with an interaction energy of ∼1.4 *k_B_T* per dNTP incorporated. In the paused state, the fork regression pressure competes for the template nucleotides, halting replication (adapted from [81]).

Under single-turnover conditions, Polγ’s correct nucleotide incorporation rate was found to be ∼30–40 s^−1^ [79,80]. However, optical tweezers single-molecule experiments (Figure 4A,B) showed that the average primer extension velocity of Polγ can vary greatly depending on the secondary structure content of the template [38]. Binding of mtSSB to the template prevents secondary structure formation, which results in an increase in the average primer extension rate of Polγ. The substantial energy required to displace an mtSSB tetramer from ssDNA (∼10 *k_B_T* per tetramer [41]) suggests that functional interactions between Polγ and mtSSB must exist. Without such interactions, the tight binding of mtSSB would be expected to impede the polymerase’s progression. Consistent with the need for Polγ to actively displace mtSSB during primer extension, optical tweezers assays revealed that the loop 2.3 region of human mtSSB (S67–L75; Figure 2B) is critical for its efficient displacement from ssDNA by Polγ [38]. Accordingly, truncation variants of mtSSB lacking loop 2.3 residues abolish the stimulatory effect of mtSSB on Polγ primer extension. Given the net negative charge of loop 2.3, it was hypothesized that repulsive electrostatic interactions between this loop and negatively charged residues on Polγ facilitate mtSSB release without compromising polymerase velocity (Figure 4C). This repulsive interaction energy was quantified at ∼12 *k_B_T* per mtSSB, which is sufficient for the polymerase to promote the release of an mtSSB tetramer from the template during primer extension activity.

#### DNA synthesis: strand displacement

As mentioned above, Polγ can unwind dsDNA or RNA–DNA hybrids to expose the template strand for replication, an activity known as strand displacement (SD) that has been extensively characterized using biochemical approaches [59,82]. Single-molecule studies have further elucidated the mechanism of DNA unwinding by Polγ and the contribution of mtSSB to this reaction (Figure 4D).

Recent optical tweezers studies shed light on the mechanism of SD by Polγ and its regulation by mtSSB [40]. Analysis of the pause-free velocities of individual replication traces (Figure 4E) revealed that Polγ itself can destabilize the first base pair of the replication fork, contributing ∼1 *k_B_T* of energy per incorporated nucleotide (Figure 4F). Binding of mtSSB to the displaced strand increases the effective fork-destabilization energy of the Polγ–mtSSB complex by 25%, reaching 1.4 *k_B_T* (Figure 4F). Given an average base-pair interaction energy of ∼3 *k_B_T*, Polγ therefore provides roughly one-third of the energy required for fork opening. This limited destabilization capacity explains the reduced velocity of the polymerase during strand displacement (∼4 bp/s) compared with primer extension (15–20 bp/s) [38]. In addition, this work found that Polγ spends a significant portion of its time in a transient inactive pause state during SD [40]. Examination of pausing as a function of the external force applied to destabilize the fork suggested that pausing is caused by competition for binding to the template between the holoenzyme and the regression pressure of the fork (Figure 4F). Loss of template engagement due to fork-regression pressure would stall polymerization and eventually favor the intramolecular kinetic partitioning of the primer between the *pol* and *exo* active sites. The observation that mtSSB binding reduces pausing during SD suggests that mtSSB counteracts fork regression pressure and stabilizes the unwound template strand [40,42] (Figure 4F). Together, these studies indicate that strand displacement by Polγ emerges from the combined action of intrinsic fork destabilization by the polymerase and stabilization of the displaced strand by mtSSB, allowing efficient replication through structured DNA regions of the mitochondrial genome.

In addition to duplex DNA, Polγ is capable of synthesizing DNA through structured templates such as G-quadruplexes. The mitochondrial genome contains multiple G-quadruplex-forming sequences, some of which may influence mtDNA replication, including motifs located near the heavy-strand replication origin (O_H_) [29,76,83,84]. The mechanism by which Polγ deals with these stable DNA structures is still to be elucidated.

#### DNA proofreading

**Figure 5:**
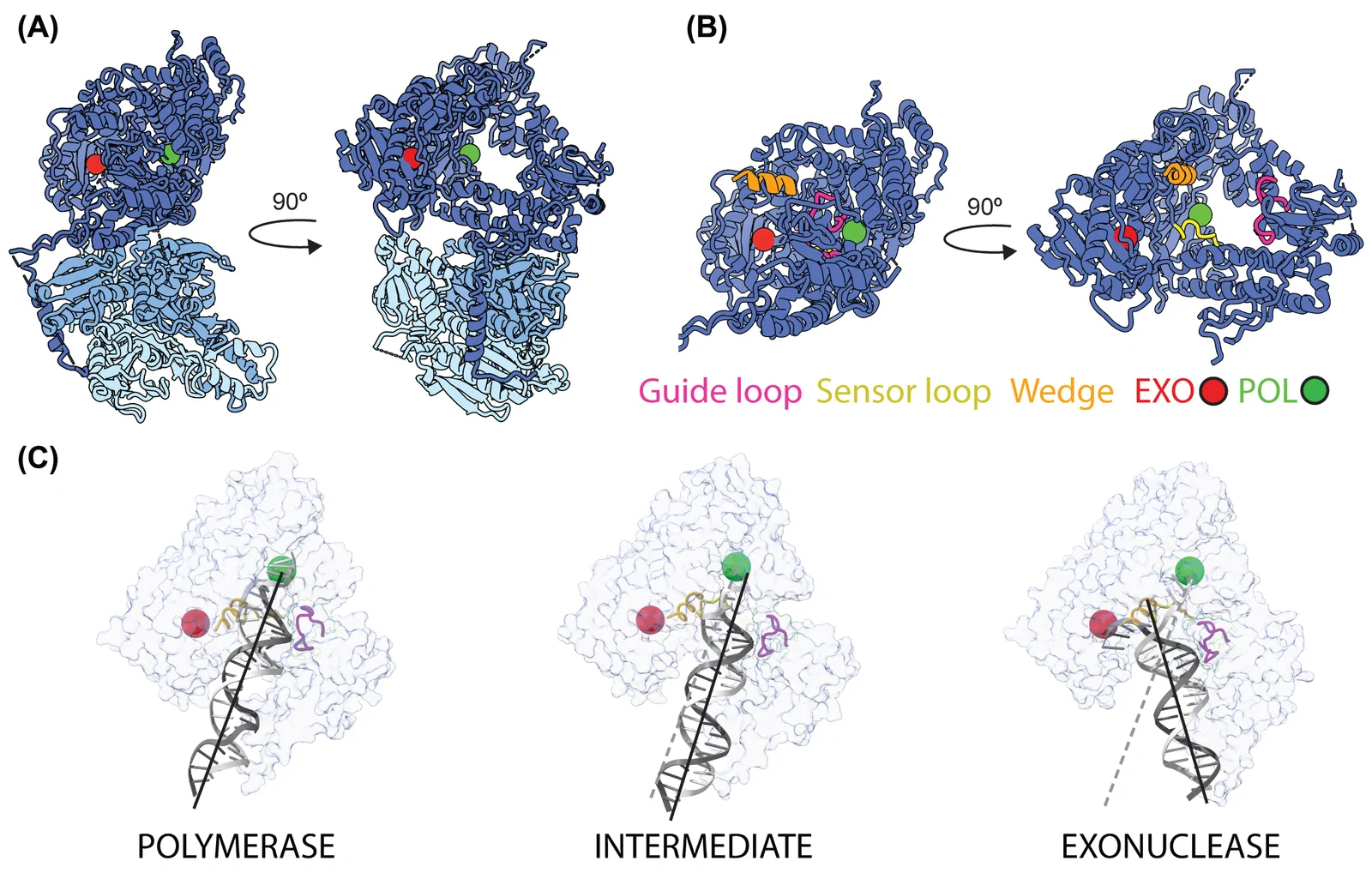
DNA proofreading by Polγ, differences in the angle of interaction between PolγA and the DNA (**A**) Position of the polymerase (green dot) and exonuclease (red dot) catalytic sites in the Polγ holoenzyme (PDB: 8D33) [61]. (**B**) PolγA model highlighting the polymerase structural regions responsible for sensing a mismatched base-pairing and later transfer to the exonuclease active site. (**C**) Polγ-DNA model showing the different orientations of the dsDNA during the pol-exo partition reaction and the positions of the polymerase (green) and exonuclease (red) catalytic sites together with the guide loop (magenta) and the wedge (yellow) subdomains. **i.** Polγ in DNA synthesis mode, with the primer strand in the polymerase catalytic site. **ii.** Polγ in an intermediate state, with the primer strand backtracked outside of the polymerase catalytic site. **iii**. Polγ in DNA exonuclease mode, with the primer strand in the exonuclease catalytic site. Primer strand, dark grey. Template strand, ligth grey Red dot: exonuclease catalytic site, green dot: polymerase catalytic site.

Polγ detects misincorporated nucleotides at the polymerase active (*pol*) site and can transfer the primer DNA strand to the exonuclease active site (*exo*) through a stepwise transfer (Figure 5A). Structural and biochemical data indicate that the enzyme switches between *pol* and *exo* modes through a non-dissociative mechanism, transferring the DNA primer intramolecularly without releasing the DNA [61,85]. Briefly, the mismatch is detected by conformational checkpoints, which sense irregular base pairing and trigger a shift from the *pol* to the *exo* mode. The enzyme then translocates along the DNA, redirecting the 3′ primer end toward the *exo* channel by the action of specific structural elements, such as the Wedge helix, Guide loop, and Sensor loop, in PolγA (Figure 5B). Repositioning of these motifs stabilizes unwound states of the primer from the template strand and guides it into the *exo* site through intermediate states (Figure 5C), in a process similar to what has been described before for other polymerases [86–89]. Within this site, a two-metal-ion-dependent hydrolytic reaction cleaves the incorrect terminal nucleotide, producing a new 3′-OH that can re-enter the polymerase site to resume DNA synthesis. Notably, in the presence of the homodimeric form of PolγB, the holoenzyme modulates the balance between polymerase and exonuclease activities, shifting the equilibrium toward DNA synthesis over degradation [85,90,91].

We hypothesize that the intramolecular transfer of the primer between active sites contributes, at least in part, to the frequent pausing observed in single-molecule studies of Polγ [38,40]. For example, see single-molecule replication traces in Figure 4. Validating this model will require single-molecule techniques with improved spatiotemporal resolution to directly correlate these transient structural transitions with pausing events.

### Replicative DNA helicase Twinkle

Twinkle is the only replicative helicase found in mitochondria. It belongs to the superfamily 4 DNA helicases, which assemble into ring-shaped oligomers that bind NTPs at the interfaces between subunits and engage approximately 12 nt of ssDNA within their central channel (∼2 nt/subunit) [35,43,92]. Twinkle provides the strand-separation activity required for leading-strand mtDNA replication. In addition, Twinkle exhibits strand-annealing, strand-exchange, and branch-migration activities and is essential for the organization of mitochondrial RNA granules [93], suggesting broader roles in mitochondrial nucleic acid metabolism. Mutations in the C10orf2 gene, encoding Twinkle, are linked to mtDNA depletion syndromes, and PEO [94,95]. Many pathogenic variants cluster in the linker region and C-terminal helicase domain, where they disrupt oligomer assembly and impair DNA unwinding, ultimately compromising replication fork progression and mtDNA copy number maintenance.

**Figure 6:**
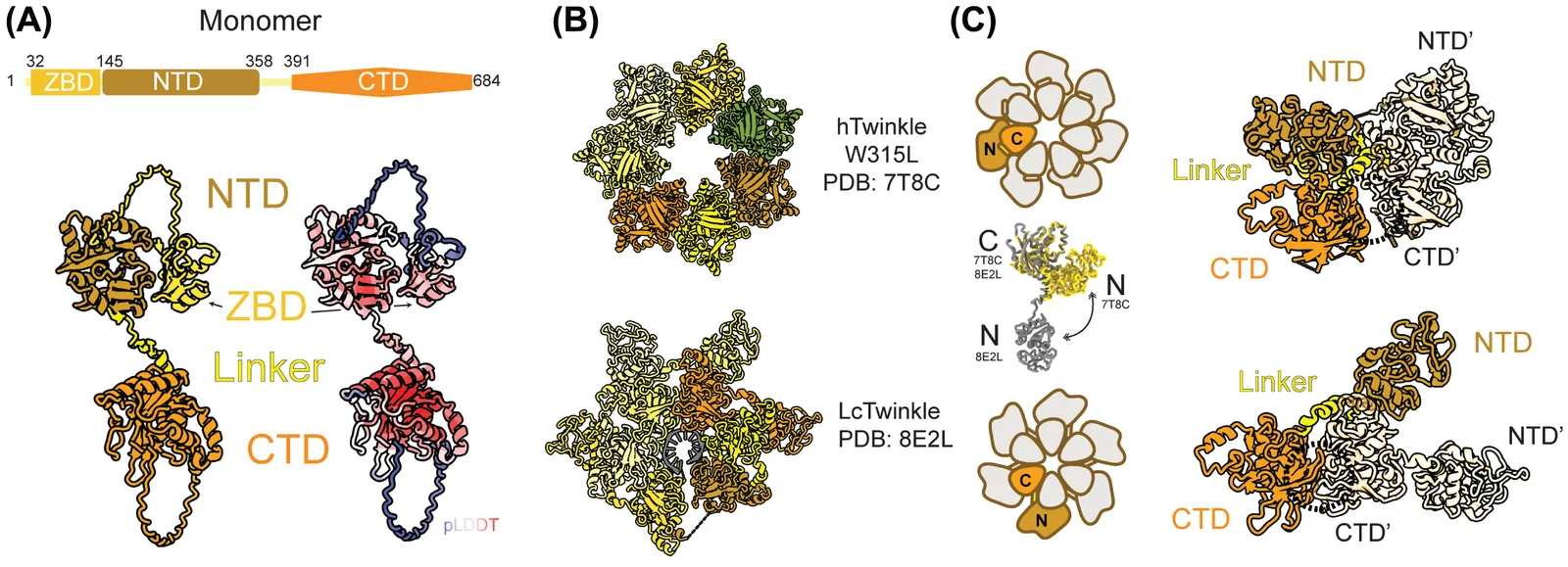
Twinkle structure and domain organization (**A**) Schematic domain organization and model of the AF3 prediction of Twinkle monomer. The ZBD, NTD, linker, and CTD are shown. (**B**) W315L-Twinkle (PDB: 7T8C) [100] and *Lc*Twinkle (PDB: 8E2L) [35] oligomeric assembly structures. (**C**) (Left) Diagrams comparing the overall organization of human W315L-Twinkle (top) and *Lc*Twinkle (bottom). (Right) Dimers of W315L-Twinkle (top) and *Lc*Twinkle (bottom) showing that in the human helicase the NTD is located over its own CTD, whereas in the fish helicase (*Lc*Twinkle), the NTD is located over the CTD of the next oligomer (marked in white).

Twinkle’s protein sequence begins with a mitochondrial localization signal, which is processed upon mitochondrial import. Although the precise cleavage point remains unknown, the field has converged on using the Δ42 variant as the mature polypeptide. Twinkle then comprises the NTD (residues 43–358), a flexible helical linker (residues 359–391), and the CTD (residues 392–684) (Figure 6). The NTD can be further divided into two different subdomains, the zinc-binding domain (ZBD) and the primase-like domain, which presents a similar fold to the primase domain of the T7 helicase homolog, but has lost its primase functions during evolution. The NTD is involved in DNA binding and oligomer organization, being essential for proper helicase activity [39,90,96,97]. The CTD of Twinkle shares high sequence similarity with that of the bacteriophage T7 gp4 helicase, including the conserved motifs responsible for helicase and ATPase activity [94,98]. The latter motifs are positioned at the interface between adjacent CTDs, allowing nucleotide binding to bridge the subunits and coordinate their movement following hydrolysis. Twinkle’s CTD terminates in an unstructured tail (C-tail) with no sequence similarity to the C-terminal tail of T7 helicase, but is well conserved among most vertebrates [99] (Figure 6). Functional studies show that deletion of this C-tail increases NTP hydrolysis activity, indicating that it acts as an intrinsic negative regulator of the helicase activity [39,96]. Notably, several point mutations in the C-tail, as well as its truncation, have been linked to human disorders, emphasizing its critical role.

#### Oligomeric state and inter-subunit contacts

One of the central questions regarding Twinkle concerns its oligomeric state. Early studies reported that Twinkle can assemble into multiple oligomeric forms, with hexamers and heptamers being the most frequently observed [94,97,101,102]. High-resolution cryo-EM structures of hexameric *Lates calcarifer* Twinkle (*LcTwinkle*) were obtained both in the apo state and bound to DNA [35], while heptameric and octameric assemblies were resolved for the human W315L-Twinkle mutant in the apo state [100], providing detailed insights into the interaction interfaces and domain architecture of the protein (Figure 6).

Upon comparison of the available Twinkle structures, a high degree of structural similarity can be observed bwtween the CTD and NTD domains. However, in the apo structure of human Twinkle bearing the W315L substitution associated with progressive external ophthalmoplegia, the CTD–NTD domains adopt a head-to-tail arrangement to form a ring [35,100], whereas in *Lc*Twinkle bound to ssDNA and ATP, the NTD is locked onto the side of the CTD of the adjacent monomer, forcing the oligomeric ring into a ‘lock-washer’ conformation. This narrow conformation is achieved by extensive contacts formed by the linker region of each monomer interacting tightly with the CTD of the neighbor (Figure 6) [35,102,100]. The transition of the NTD between these positions appears to be regulated by the presence of nucleotide triphosphates [103]. Finally, cryo-EM maps of *Lc*Twinkle bound to DNA revealed that the CTD faces the replication fork, while the NTD swings ∼90° to the side, with the primase-like domain oriented downward, preventing contact with the translocating DNA strand (Figure 6C). While the precise molecular implications of these alternative NTD arrangements remain unclear, they are likely to play a key role in Twinkle regulation and oligomerization, influencing both DNA interaction and the dynamic architecture of the helicase.

Moreover, single-molecule approaches, which are not constrained by the structural stability requirements of cryo-EM, have revealed an even broader oligomeric heterogeneity. AFM studies identified populations ranging from monomers to hexamers and higher-order oligomers [104], whereas optical tweezers combined with confocal microscopy enabled direct visualization of multiple oligomeric states of eGFP–Twinkle bound to individual DNA molecules [39]. The functional significance of these different oligomeric states remains unclear. However, in the absence of a dedicated helicase loader, it is tempting to speculate that lower-order oligomers facilitate direct self-loading onto DNA, followed by completion of hexameric or heptameric assembly to form the active helicase.

#### Loading on DNA

**Figure 7:**
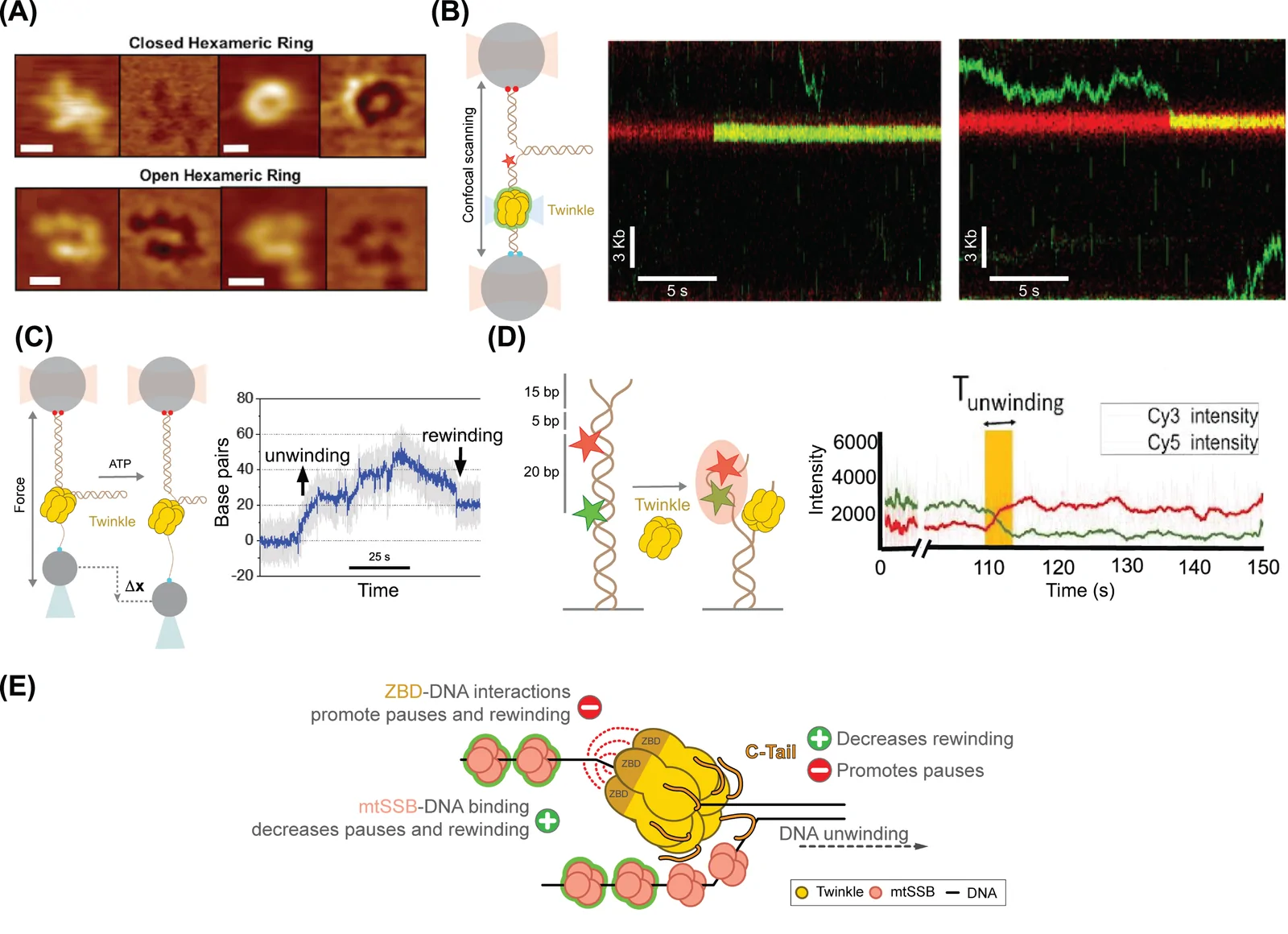
Single-molecule studies on Twinkle DNA loading and unwinding kinetics (**A**) AFM imaging revealed that Twinkle exists in both closed-ring and open-ring configurations. Scale bars, 25 nm. Adapted from Kaur et al. [104]. (**B**) (*Left*) Diagram of the optical tweezers-confocal assay to image eGFP–Twinkle (green) on individual DNA constructs containing a DNA fork attached between optically trapped beads. The fork is labeled with Atto 647N (red star). (*Right*) Representative kymographs of eGFP–Twinkle oligomers (green) binding directly (left) or diffusing before loading (right) at the fork position (in red) (adapted from [39]). (**C**) (*Left*) Schematic of optical trapping assays to measure Twinkle real-time unwinding kinetics. A DNA fork-like construct is tethered between a bead held in the optical trap (red cone) and a bead on a micropipette (light blue). DNA unwinding is recorded as a change in tether extension (Δx) under constant mechanical force. (*Right*) Representative trace showing Twinkle DNA unwinding (increase in bp) and rewinding (decrease in bp) activities (adapted from [39]). (**D**) (Left) Schematic of the smFRET assay. A forked dsDNA with two 15-nt ssDNA overhangs was anchored on a PEG-covered slide and labeled with a pair of Cy3 (green)-Cy5 (red) fluorophores. (Right) The stepwise increase in the FRET value time trajectory indicates stepwise dsDNA unwinding by Twinkle (adapted from [105]). (**E**) Model for mtSSB relief of ZBD- and C-tail-mediated inhibition of Twinkle activity. During DNA unwinding, ZBD-DNA interactions work as a molecular brake, promoting pauses and rewinding events. During this reaction, the C-tail down-regulates ATP turnover, leading to additional pauses, further reducing the DNA unwinding rate. mtSSB binding to the DNA outcompetes the inhibitory ZBD-DNA interactions, enhancing functional loading, decreasing pauses during DNA unwinding, and reducing the probability of rewinding events.

Loading of Twinkle at the replication fork is a critical regulatory step for initiating DNA replication. However, no helicase loader has been found in mitochondria. Cryo-EM studies have shown conformational flexibility that could help enable helicase loading by a ring-opening mechanism [35,100]. In support of this model, AFM and HS-AFM with *Hs*Twinkle and *Lc*Twinkle, respectively, have shown that Twinkle ring-like oligomers can switch between closed and open conformations, allowing them to load (and unload) onto DNA without the need for a dedicated helicase loader [35,104] (Figure 7A). Furthermore, smFRET data showed that increasing Twinkle protein concentration enhanced unwinding initiation but not the subsequent unwinding rate [105], suggesting a possible assembly pathway involving stepwise recruitment of Twinkle subunits at the fork.

Optical tweezers combined with confocal microscopy recently showed that Twinkle (labeled with eGFP) can load and undergo one-dimensional diffusion on dsDNA, reaching diffusion rates of thousands of base pairs per second (Figure 7B) [39]. Notably, reaching the fork halts diffusion, suggesting that Twinkle forms a stable complex, an event likely driven by its simultaneous engagement with both DNA strands. This process may facilitate efficient localization of replication forks within the ∼16 kbp mitochondrial genome [39].

#### DNA unwinding kinetics

FRET and optical tweezers studies showed that, once functionally loaded, Twinkle unwinds DNA at average velocities ranging from ∼0.7 to 4 bp/s as the ATP concentration increases from 1 to 10 mM [39,104,105]. These velocities are relatively slow compared with those expected for the related bacteriophage T7 helicase and *E. coli* DnaB in the absence of assisting force, which reach ∼25 and ∼50 bp/s, respectively [106,107].

Optical tweezers studies showed that the ZBD and C-tail of Twinkle down-regulate the unwinding kinetics of the helicase by significantly decreasing the average velocity [39,104,105]. On the one hand, the flexible and dynamic nature of the NTD, where the ZBD is located, may facilitate regulatory interactions with the two ssDNA strands at the replication fork [108]. These interactions could act as a ‘molecular brake’ that transiently stalls helicase progression. On the other hand, the unstructured C-tail acts as a negative regulator by limiting ATP turnover [99], which consequently reduces helicase velocity and increases the frequency of pause events (Figure 7E).

In addition, structural studies of *Lc*Twinkle showed that all ATP-binding sites adopt equivalent conformations. This arrangement suggests that ATP hydrolysis may occur with similar probabilities across subunits, potentially resulting in uncoordinated and nonproductive hydrolysis events when replisome partners are absent from the fork [35]. This hypothesis was also supported by smFRET studies on human Twinkle (Figure 7D) [105]. This uncoordinated, probabilistic mode of ATP hydrolysis contrasts with the canonical, sequential (‘hand-over-hand’) mechanism established for hexameric helicases and translocases such as papillomavirus E1 and bacterial Rho [109,110]. In these systems, distinct conformational states along the ring impose sequential ATP turnover, thereby driving continuous and coordinated translocation. The lack of comparable structural asymmetry in Twinkle may promote transient inactive or paused states that reduce its average unwinding velocity.

Single-molecule studies further demonstrated that both unwinding rates and processivity are enhanced by mtSSB [39,104]. Mechanistically, this stimulation was attributed to a reduction in pausing, as mtSSB outcompetes the ZBD for ssDNA binding, effectively releasing the ZBD’s restrictive regulatory effect on unwinding kinetics (Figure 7E) [39,105]. The stimulation was observed to be specific to mtSSB, ruling out models in which stimulation arises simply from passive coating of newly generated ssDNA by mtSSB, which may otherwise facilitate fraying of the fork.

smFRET and optical tweezers experiments also revealed that Twinkle unwinding activity is generally followed by partial or total rewinding transitions (Figure 7C,D). Rewinding activity is mediated by a single Twinkle oligomer rather than by passive DNA rezipping [108] and occurs independently of ATP concentration. Rewinding was inhibited by binding of mtSSB to the displaced strand and/or deletion of Twinkle’s zinc-binding domain (ΔZBD), which is involved in ssDNA binding. These results suggest that interactions of Twinkle with the displaced strand play an essential role in the rewinding reaction [39,105]. While the requirements for rewinding resemble those of Twinkle’s ‘intermolecular’ DNA annealing activity [92,111], the two processes may be mechanistically different.

Together, structural and single-molecule studies indicate that Twinkle activity is regulated by a combination of oligomeric plasticity, domain-mediated autoregulation, and interactions with mtSSB, enabling the helicase to adapt its activity to the dynamic environment of the mitochondrial replication fork.

### Outlook

#### Toward a complete structural and mechanistic model of the mitochondrial replisome

A complete structural and mechanistic model of the mitochondrial replisome remains a central objective in the field. Significant progress has been achieved through high-resolution structures of Polγ and recent cryo-EM reconstructions of mtSSB and Twinkle assemblies. However, atomic-resolution structures of the native human Twinkle hexamer in complex with a replication-fork substrate, and of the complete mitochondrial replisome, remain major missing pieces. Obtaining such structures will be particularly challenging because the mitochondrial replisome is likely to form a pleomorphic and dynamic ensemble, in which its components adopt multiple arrangements and engage in transient interactions as DNA synthesis proceeds. Nevertheless, resolving these assemblies will be essential for understanding how helicase and polymerase activities are coordinated at the mitochondrial replication fork.

Structural advances have been complemented by single-molecule studies that provide quantitative models of the kinetic behavior of individual replisome components. Intriguingly, both Polγ and Twinkle exhibit extensive pausing during fork progression, even in the presence of mtSSB. This raises an important question: does the replisome ensemble operate through synergistic coupling that mitigates the intrinsic inefficiencies of individual enzymes, or do additional regulatory mechanisms or factors contribute to maintaining physiological replication rates? Addressing this will require experiments that can simultaneously track multiple replisome components during DNA synthesis.

#### Reconstitution of mitochondrial DNA replication

Despite decades of biochemical investigation, a fully processive *in vitro* reconstitution of mtDNA replication on a physiologically relevant circular substrate remains an important milestone. While rolling-circle assays have provided valuable insight into the coordinated activities of Polγ, Twinkle, and mtSSB, to our knowledge, processive replication over a double-stranded circular DNA substrate has not yet been achieved. Achieving such a system would allow direct investigation of how polymerase, helicase, and ssDNA-binding proteins coordinate processive DNA synthesis at native mitochondrial sequences and fork structures.

#### Sequence context and DNA topology

An additional challenge is understanding how sequence context and DNA topology influence replication dynamics across the mitochondrial genome. Emerging sequencing approaches, including nanopore-based detection of replication intermediates, offer the possibility of mapping pause sites and structural barriers such as G-quadruplexes directly *in vivo*. These approaches may also help clarify the regulation of replication at specific genomic elements such as the termination-associated sequence (TAS) [112], where replication pausing and fork arrest have long been observed but remain poorly understood. Early work suggested that local fluctuations in Twinkle abundance may influence processive replication across this region, yet the molecular determinants controlling helicase recruitment or activity at TAS remain unidentified [113,114]. At the same time, technological advances in single-molecule approaches will provide higher spatial and temporal resolution, enabling precise kinetic analysis of sequence-dependent effects on replication dynamics. Integrating these genomic insights with quantitative biophysical models of polymerase and helicase activity may clarify how sequence dependencies shape replication fork progression and stability in mitochondria.

#### Implications for mitochondrial disease

Finally, a quantitative mechanistic understanding of mitochondrial replisome function will be essential for interpreting the molecular basis of mitochondrial disease. Mutations in POLG and C10orf2 disrupt replisome activity and cause a wide spectrum of mtDNA maintenance disorders (e.g., Alper’s and Perrault’s syndromes, respectively). Integrating structural, biochemical, and single-molecule insights should help clarify how pathogenic variants perturb the energetic balance between DNA synthesis, pausing, and proofreading. Such mechanistic understanding will also provide a framework for therapeutic strategies aimed at stabilizing replisome function, as illustrated by recent efforts to pharmacologically modulate Polγ activity [8].

## Open Access

Open access for this article was enabled by the participation of the Unidad Asociada al Centro Nacional de Biotecnología in an all-inclusive Read & Publish agreement with Portland Press and the Biochemical Society under an agreement with CSIC.

## Abbreviations

AID: accessory-interacting determinant
CTD: C-terminal domain
cryo-EM: cryo-electron microscopy
D-loop: displacement loop
dsDNA: double-stranded DNA
*Eco*SSB: *Escherichia coli* SSB
Exo: exonuclease
HLH-β3: helix–loop–helix β3 dimerization
*Lc*Twinkle: *Lates calcarifer* Twinkle
mtDNA: mitochondrial DNA
mtSSB: mitochondrial single-stranded DNA-binding protein
NTD: N-terminal domain
OB: oligonucleotide/oligosaccharide-binding
PEO: progressive external ophthalmoplegia
Pol: polymerase
Polγ: polymerase γ
SD: strand displacement
ssDNA: single-stranded DNA
TAS: termination-associated sequence
ZBC: zinc-bindng domain

## References

[1] Anderson S., Bankier A.T., Barrell B.G., de Bruijn M.H., Coulson A.R., Drouin J. et al. (1981) Sequence and organization of the human mitochondrial genome. Nature 290, 457–465 10.1038/290457a0 7219534

[2] DiMauro S. and Schon E.A. (2003) Mitochondrial respiratory-chain diseases. N. Engl. J. Med. 348, 2656–2668 10.1056/NEJMra022567 12826641

[3] Wallace D.C. (2010) Mitochondrial DNA mutations in disease and aging. Environ. Mol. Mutagen. 51, 440–450 10.1002/em.20586 20544884

[4] Faridi R., Rea A., Fenollar-Ferrer C., O'Keefe R.T., Gu S., Munir Z. et al. (2022) New insights into Perrault syndrome, a clinically and genetically heterogeneous disorder. Hum. Genet. 141, 805–819 10.1007/s00439-021-02319-7 34338890 PMC11330641

[5] Copeland W.C. (2012) Defects in mitochondrial DNA replication and human disease. Crit. Rev. Biochem. Mol. Biol. 47, 64–74 10.3109/10409238.2011.632763 22176657 PMC3244805

[6] Gorman G.S., Chinnery P.F., DiMauro S., Hirano M., Koga Y., McFarland R. et al. (2016) Mitochondrial diseases. Nat. Rev. Dis Primers 2, 16080 10.1038/nrdp.2016.80 27775730

[7] Lagouge M. and Larsson N.-G. (2013) The role of mitochondrial DNA mutations and free radicals in disease and ageing. J. Intern. Med. 273, 529–543 10.1111/joim.12055 23432181 PMC3675642

[8] Valenzuela S., Zhu X., Macao B., Stamgren M., Geukens C., Charifson P.S. et al. (2025) Small molecules restore mutant mitochondrial DNA polymerase activity. Nature 642, 501–507 10.1038/s41586-025-08856-9 40205042 PMC12158775

[9] Copeland B. (2026) Human DNA Polymerase Gamma Mutation Database. National Institute of Environmental Health Sciences[cited 2026 Aug 7] Available from: https://portal.niehs.nih.gov/polg/

[10] Suomalainen A. and Battersby B.J. (2018) Mitochondrial diseases: the contribution of organelle stress responses to pathology. Nat. Rev. Mol. Cell Biol. 19, 77–92 10.1038/nrm.2017.66 28792006

[11] Calvo S.E. and Mootha V.K. (2010) The mitochondrial proteome and human disease. Annu. Rev. Genomics Hum. Genet. 11, 25–44 10.1146/annurev-genom-082509-141720 20690818 PMC4397899

[12] Ciesielski G.L., Oliveira M.T. and Kaguni L.S. (2016) Animal mitochondrial DNA replication. Enzymes 39, 255–292 10.1016/bs.enz.2016.03.006 27241933 PMC4964852

[13] Bernardino Gomes T.M., Vincent A.E., Menger K.E., Stewart J.B. and Nicholls T.J. (2024) Mechanisms and pathologies of human mitochondrial DNA replication and deletion formation. Biochem. J. 481, 683–715 10.1042/BCJ20230262 38804971 PMC11346376

[14] Rahman S. and Copeland W.C. (2019) POLG-related disorders and their neurological manifestations. Nat. Rev. Neurol 15, 40–52 10.1038/s41582-018-0101-0 30451971 PMC8796686

[15] Peter B. and Falkenberg M. (2020) TWINKLE and other human mitochondrial DNA helicases: structure, function and disease. Genes. 11, 408 10.3390/genes11040408 32283748 PMC7231222

[16] Del Dotto V., Ullah F., Di Meo I., Magini P., Gusic M., Maresca A. et al. (2020) SSBP1 mutations cause mtDNA depletion underlying a complex optic atrophy disorder. J. Clin. Invest. 130, 108–125 10.1172/JCI128514 31550240 PMC6934201

[17] Nicholls T.J. and Minczuk M. (2014) In D-loop: 40 years of mitochondrial 7S DNA. Exp. Gerontol. 56, 175–181 10.1016/j.exger.2014.03.027 24709344

[18] Brown T.A., Cecconi C., Tkachuk A.N., Bustamante C. and Clayton D.A. (2005) Replication of mitochondrial DNA occurs by strand displacement with alternative light-strand origins, not via a strand-coupled mechanism. Genes Dev. 19, 2466–2476 10.1101/gad.1352105 16230534 PMC1257401

[19] Miralles Fusté J., Shi Y., Wanrooij S., Zhu X., Jemt E., Persson Ö. et al. (2014) *In vivo* occupancy of mitochondrial single-stranded DNA binding protein supports the strand displacement mode of DNA replication. PLos Genet. 10, e1004832 10.1371/journal.pgen.1004832 25474639 PMC4256270

[20] Reyes A., Kazak L., Wood S.R., Yasukawa T., Jacobs H.T. and Holt I.J. (2013) Mitochondrial DNA replication proceeds via a ‘bootlace’ mechanism involving the incorporation of processed transcripts. Nucleic Acids Res. 41, 5837–5850 10.1093/nar/gkt196 23595151 PMC3675460

[21] Jemt E., Persson Ö., Shi Y., Mehmedovic M., Uhler J.P., Dávila López M. et al. (2015) Regulation of DNA replication at the end of the mitochondrial D-loop involves the helicase TWINKLE and a conserved sequence element. Nucleic Acids Res. 43, 9262–9275 10.1093/nar/gkv804 26253742 PMC4627069

[22] Clayton D.A. (1982) Replication of animal mitochondrial DNA. Cell 28, 693–705 10.1016/0092-8674(82)90049-6 6178513

[23] Holt I.J. and Jacobs H.T. (2014) Unique features of DNA replication in mitochondria: a functional and evolutionary perspective: insights & perspectives. Bioessays 36, 1024–1031 10.1002/bies.201400052 25220172

[24] Yasukawa T., Reyes A., Cluett T.J., Yang M.-Y., Bowmaker M., Jacobs H.T. et al. (2006) Replication of vertebrate mitochondrial DNA entails transient ribonucleotide incorporation throughout the lagging strand. EMBO J. 25, 5358–5371 10.1038/sj.emboj.7601392 17066082 PMC1636616

[25] Singh A., Johnson L.C., Baruch-Torres N., Yin Y.W. and Patel S.S. (2026) Human mitochondrial helicase Twinkle has RNA binding, annealing, and strand-exchange activities. Nucleic Acids Res. 54, gkag008 10.1093/nar/gkag008 41562253 PMC12820530

[26] Wanrooij S., Miralles Fusté J., Stewart J.B., Wanrooij P.H., Samuelsson T., Larsson N.-G. et al. (2012) *In vivo* mutagenesis reveals that OriL is essential for mitochondrial DNA replication. EMBO Rep. 13, 1130–1137 10.1038/embor.2012.161 23090476 PMC3513414

[27] Roos S., Macao B., Fusté J.M., Lindberg C., Jemt E., Holme E. et al. (2013) Subnormal levels of POLγA cause inefficient initiation of light-strand DNA synthesis and lead to mitochondrial DNA deletions and progressive external ophthalmoplegia [corrected]. Hum. Mol. Genet. 22, 2411–2422 10.1093/hmg/ddt094 23446635

[28] Wanrooij S., Goffart S., Pohjoismäki J.L.O., Yasukawa T. and Spelbrink J.N. (2007) Expression of catalytic mutants of the mtDNA helicase Twinkle and polymerase POLG causes distinct replication stalling phenotypes. Nucleic Acids Res. 35, 3238–3251 10.1093/nar/gkm215 17452351 PMC1904276

[29] Dong D.W., Pereira F., Barrett S.P., Kolesar J.E., Cao K., Damas J. et al. (2014) Association of G-quadruplex forming sequences with human mtDNA deletion breakpoints. BMC Genomics 15, 677 10.1186/1471-2164-15-677 25124333 PMC4153896

[30] Persson Ö., Muthukumar Y., Basu S., Jenninger L., Uhler J.P., Berglund A.-K. et al. (2019) Copy-choice recombination during mitochondrial L-strand synthesis causes DNA deletions. Nat. Commun. 10, 759 10.1038/s41467-019-08673-5 30770810 PMC6377680

[31] Oliveira M.T., Pontes de C.B. and Ciesielski G.L. (2020) Roles of the mitochondrial replisome in mitochondrial DNA deletion formation. Genet. Mol. Biol. 43, e20190069 10.1590/1678-4685-gmb-2019-0069 32141473 PMC7197994

[32] Holt I.J., Lorimer H.E. and Jacobs H.T. (2000) Coupled leading- and lagging-strand synthesis of mammalian mitochondrial DNA. Cell 100, 515–524 10.1016/S0092-8674(00)80688-1 10721989

[33] Falkenberg M., Larsson N.-G. and Gustafsson C.M. (2024) Replication and transcription of human mitochondrial DNA. Annu. Rev. Biochem. 93, 47–77 10.1146/annurev-biochem-052621-092014 38594940

[34] Phillips A.F., Millet A.R., Tigano M., Dubois S.M., Crimmins H., Babin L. et al. (2017) Single-molecule analysis of mtDNA replication uncovers the basis of the common deletion. Mol. Cell 65, 527.e6–538.e6 10.1016/j.molcel.2016.12.014 28111015

[35] Li Z., Kaur P., Lo C.-Y., Chopra N., Smith J., Wang H. et al. (2022) Structural and dynamic basis of DNA capture and translocation by mitochondrial Twinkle helicase. Nucleic Acids Res. 50, 11965–11978 10.1093/nar/gkac1089 36400570 PMC9723800

[36] Jiang M., Xie X., Zhu X., Jiang S., Milenkovic D., Misic J. et al. (2021) The mitochondrial single-stranded DNA binding protein is essential for initiation of mtDNA replication. Sci. Adv. 7, 10.1126/sciadv.abf8631 PMC11057760 34215584

[37] Takamatsu C., Umeda S., Ohsato T., Ohno T., Abe Y., Fukuoh A. et al. (2002) Regulation of mitochondrial D-loops by transcription factor A and single-stranded DNA-binding protein. EMBO Rep. 3, 451–456 10.1093/embo-reports/kvf099 11964388 PMC1084112

[38] Cerrón F., de Lorenzo S., Lemishko K.M., Ciesielski G.L., Kaguni L.S., Cao F.J. et al. (2019) Replicative DNA polymerases promote active displacement of SSB proteins during lagging strand synthesis. Nucleic Acids Res. 47, 5723–5734 10.1093/nar/gkz249 30968132 PMC6582349

[39] Plaza-G.A I., Ortiz-Rodríguez M., Buchanan S.P., Miguez-Amil S., Lemishko K.M., Moreno-Herrero F. et al. (2025) Autoregulation of the real-time kinetics of the human mitochondrial replicative helicase. Nat. Commun. 16, 5460 10.1038/s41467-025-60289-0 40592829 PMC12214949

[40] Plaza-G.A I., Lemishko K.M., Crespo R., Truong T.Q., Kaguni L.S., Cao-García F.J. et al. (2023) Mechanism of strand displacement DNA synthesis by the coordinated activities of human mitochondrial DNA polymerase and SSB. Nucleic Acids Res. 51, 1750–1765 10.1093/nar/gkad037 36744436 PMC9976888

[41] Morin J.A., Cerrón F., Jarillo J., Beltran-Heredia E., Ciesielski G.L., Arias-Gonzalez J.R. et al. (2017) DNA synthesis determines the binding mode of the human mitochondrial single-stranded DNA-binding protein. Nucleic Acids Res. 45, 7237–7248 10.1093/nar/gkx395 28486639 PMC5499585

[42] Ciesielski G.L., Bermek O., Rosado-Ruiz F.A., Hovde S.L., Neitzke O.J., Griffith J.D. et al. (2015) Mitochondrial single-stranded DNA-binding proteins stimulate the activity of DNA polymerase γ by organization of the template DNA. J. Biol. Chem. 290, 28697–28707 10.1074/jbc.M115.673707 26446790 PMC4661385

[43] Korhonen J.A., Gaspari M. and Falkenberg M. (2003) TWINKLE has 5′ → 3′ DNA helicase activity and is specifically stimulated by mitochondrial single-stranded DNA-binding protein*. J. Biol. Chem. 278, 48627–48632 10.1074/jbc.M306981200 12975372

[44] Riccio A.A., Bouvette J., Pedersen L.C., Somai S., Dutcher R.C., Borgnia M.J. et al. (2024) Structures of the mitochondrial single-stranded DNA binding protein with DNA and DNA polymerase γ. Nucleic Acids Res. 52, 10329–10340 10.1093/nar/gkae670 39106165 PMC11417365

[45] Ciesielski G.L., Rosado-Ruiz F.A. and Kaguni L.S. (2016) Purification and comparative assay of human mitochondrial single-stranded DNA-binding protein. Methods Mol. Biol. 1351, 211–222 10.1007/978-1-4939-3040-1_16 26530685 PMC4703105

[46] Wu D., Kaur P., Li Z.M., Bradford K.C., Wang H. and Erie D.A. (2016) Visualizing the path of DNA through proteins using DREEM imaging. Mol. Cell 61, 315–323 10.1016/j.molcel.2015.12.012 26774284 PMC5242388

[47] Yang C., Curth U., Urbanke C. and Kang C. (1997) Crystal structure of human mitochondrial single-stranded DNA binding protein at 2.4 Å resolution. Nat. Struct. Biol. 4, 153–157 10.1038/nsb0297-153 9033597

[48] Raghunathan S., Ricard C.S., Lohman T.M. and Waksman G. (1997) Crystal structure of the homo-tetrameric DNA binding domain of *Escherichia coli* single-stranded DNA-binding protein determined by multiwavelength x-ray diffraction on the selenomethionyl protein at 2.9-A resolution. Proc. Natl. Acad. Sci. U.S.A. 94, 6652–6657 10.1073/pnas.94.13.6652 9192620 PMC21213

[49] Piro-Mégy C., Sarzi E., Tarrés-Solé A., Péquignot M., Hensen F., Quilès M. et al. (2020) Dominant mutations in mtDNA maintenance gene SSBP1 cause optic atrophy and foveopathy. J. Clin. Invest. 130, 143–156 10.1172/JCI128513 31550237 PMC6934222

[50] Martucci M., Moretton A., Tarrés-Solé A., Ropars V., Lambert L., Vernet P. et al. (2024) The mutation R107Q alters mtSSB ssDNA compaction ability and binding dynamics. Nucleic Acids Res. 52, 5912–5927 10.1093/nar/gkae354 38742632 PMC11162770

[51] Qian Y. and Johnson K.A. (2017) The human mitochondrial single-stranded DNA-binding protein displays distinct kinetics and thermodynamics of DNA binding and exchange. J. Biol. Chem. 292, 13068–13084 10.1074/jbc.M117.791392 28615444 PMC5546044

[52] Ciesielski G.L., Kim S., de Bovi Pontes C. and Kaguni L.S. (2021) Physical and functional interaction of mitochondrial single-stranded DNA-binding protein and the catalytic subunit of DNA polymerase gamma. Front. Genet 12, 721864 10.3389/fgene.2021.721864 34539752 PMC8440931

[53] Lohman T.M. and Overman L.B. (1985) Two binding modes in *Escherichia coli* single strand binding protein-single stranded DNA complexes. Modulation by NaCl concentration. J. Biol. Chem. 260, 3594–3603 10.1016/S0021-9258(19)83663-3 3882711

[54] Lohman T.M. and Ferrari M.E. (1994) *Escherichia coli* single-stranded DNA-binding protein: multiple DNA-binding modes and cooperativities. Annu. Rev. Biochem. 63, 527–570 10.1146/annurev.bi.63.070194.002523 7979247

[55] Lohman T.M., Bujalowski W. and Overman L.B. (1988) E. coli single strand binding protein: a new look at helix-destabilizing proteins. Trends Biochem. Sci. 13, 250–255 2855682

[56] Czernecki D., Nourisson A., Legrand P. and Delarue M. (2023) Reclassification of family A DNA polymerases reveals novel functional subfamilies and distinctive structural features. Nucleic Acids Res. 51, 4488–4507 10.1093/nar/gkad242 37070157 PMC10201439

[57] Fan L., Kim S., Farr C.L., Schaefer K.T., Randolph K.M., Tainer J.A. et al. (2006) A novel processive mechanism for DNA synthesis revealed by structure, modeling and mutagenesis of the accessory subunit of human mitochondrial DNA polymerase. J. Mol. Biol. 358, 1229–1243 10.1016/j.jmb.2006.02.073 16574152 PMC4703138

[58] Carrodeguas J.A., Theis K., Bogenhagen D.F. and Kisker C. (2001) Crystal structure and deletion analysis show that the accessory subunit of mammalian DNA polymerase g, PolgB, functions as a homodimer. Mol. Cell 7, 43–54 10.1016/S1097-2765(01)00153-8 11172710

[59] Corrà S., Zuppardo A., Valenzuela S., Jenninger L., Cerutti R., Sillamaa S. et al. (2025) Modelling POLG mutations in mice unravels a critical role of POLγΒ in regulating phenotypic severity. Nat. Commun. 16, 1–18 10.1038/s41467-025-60059-y 40404629 PMC12098916

[60] Trifunovic A., Wredenberg A., Falkenberg M., Spelbrink J.N., Rovio A.T., Bruder C.E. et al. (2004) Premature ageing in mice expressing defective mitochondrial DNA polymerase. Nature 429, 417–423 10.1038/nature02517 15164064

[61] Park J., Herrmann G.K., Mitchell P.G., Sherman M.B. and Yin Y.W. (2023) Polγ coordinates DNA synthesis and proofreading to ensure mitochondrial genome integrity. Nat. Struct. Mol. Biol. 30, 812–823 10.1038/s41594-023-00980-2 37202477 PMC10920075

[62] Longley M.J., Prasad R., Srivastava D.K., Wilson S.H. and Copeland W.C. (1998) Identification of 5′-deoxyribose phosphate lyase activity in human DNA polymerase gamma and its role in mitochondrial base excision repair *in vitro*. Proc. Natl. Acad. Sci. U S.A. 95, 12244–12248 10.1073/pnas.95.21.12244 9770471 PMC22816

[63] Lee Y.-S., Kennedy W.D. and Yin Y.W. (2009) Structural insight into processive human mitochondrial DNA synthesis and disease-related polymerase mutations. Cell 139, 312–324 10.1016/j.cell.2009.07.050 19837034 PMC3018533

[64] Lim S.E., Longley M.J. and Copeland W.C. (1999) The mitochondrial p55 accessory subunit of human DNA polymerase gamma enhances DNA binding, promotes processive DNA synthesis, and confers *N*-ethylmaleimide resistance. J. Biol. Chem. 274, 38197–38203 10.1074/jbc.274.53.38197 10608893

[65] Johnson A.A., Tsai Y.c., Graves S.W. and Johnson K.A. (2000) Human mitochondrial DNA polymerase holoenzyme: reconstitution and characterization. Biochemistry 39, 1702–1708 10.1021/bi992104w 10677218

[66] Nurminen A., Farnum G.A. and Kaguni L.S. (2017) Pathogenicity in POLG syndromes: DNA polymerase gamma pathogenicity prediction server and database. BBA Clin. 7, 147–156 10.1016/j.bbacli.2017.04.001 28480171 PMC5413197

[67] Sohl C.D., Szymanski M.R., Mislak A.C., Shumate C.K., Amiralaei S., Schinazi R.F. et al. (2015) Probing the structural and molecular basis of nucleotide selectivity by human mitochondrial DNA polymerase γ. Proc. Natl. Acad. Sci. U.S.A. 112, 8596–8601 10.1073/pnas.1421733112 26124101 PMC4507203

[68] Szymanski M.R., Kuznetsov V.B., Shumate C., Meng Q., Lee Y.-S., Patel G. et al. (2015) Structural basis for processivity and antiviral drug toxicity in human mitochondrial DNA replicase. EMBO J. 34, 1959–1970 10.15252/embj.201591520 26056153 PMC4547898

[69] Di Re M., Sembongi H., He J., Reyes A., Yasukawa T., Martinsson P. et al. (2009) The accessory subunit of mitochondrial DNA polymerase gamma determines the DNA content of mitochondrial nucleoids in human cultured cells. Nucleic Acids Res. 37, 5701–5713 10.1093/nar/gkp614 19625489 PMC2761280

[70] Wojtaszek J.L., Hoff K.E., Longley M.J., Kaur P., Andres S.N., Wang H. et al. (2023) Structure-specific roles for PolG2-DNA complexes in maintenance and replication of mitochondrial DNA. Nucleic Acids Res. 51, 9716–9732 10.1093/nar/gkad679 37592734 PMC10570022

[71] Johnson A.A. and Johnson K.A. (2001) Fidelity of nucleotide incorporation by human mitochondrial DNA polymerase. J. Biol. Chem. 276, 38090–38096 10.1074/jbc.M106045200 11477093

[72] Kaguni L.S. (2004) DNA polymerase gamma, the mitochondrial replicase. Annu. Rev. Biochem. 73, 293–320 10.1146/annurev.biochem.72.121801.161455 15189144

[73] Kunkel T.A. and Soni A. (1988) Exonucleolytic proofreading enhances the fidelity of DNA synthesis by chick embryo DNA polymerase-gamma. J. Biol. Chem. 263, 4450–4459 10.1016/S0021-9258(18)68947-1 2831231

[74] Longley M.J., Nguyen D., Kunkel T.A. and Copeland W.C. (2001) The fidelity of human DNA polymerase gamma with and without exonucleolytic proofreading and the p55 accessory subunit. J. Biol. Chem. 276, 38555–38562 10.1074/jbc.M105230200 11504725

[75] Pinz K.G. and Bogenhagen D.F. (2000) Characterization of a catalytically slow AP lyase activity in DNA polymerase gamma and other family A DNA polymerases. J. Biol. Chem. 275, 12509–12514 10.1074/jbc.275.17.12509 10777538

[76] Sullivan E.D., Longley M.J. and Copeland W.C. (2020) Polymerase γ efficiently replicates through many natural template barriers but stalls at the HSP1 quadruplex. J. Biol. Chem. 295, 17802–17815 10.1074/jbc.RA120.015390 33454015 PMC7762954

[77] Seshadri A. and Badrinarayanan A. (2025) Exonuclease action of replicative polymerase gamma drives damage-induced mitochondrial DNA clearance. EMBO Rep. 26, 1385–1405 10.1038/s44319-025-00380-1 39890960 PMC11894172

[78] Nissanka N., Bacman S.R., Plastini M.J. and Moraes C.T. (2018) The mitochondrial DNA polymerase gamma degrades linear DNA fragments precluding the formation of deletions. Nat. Commun. 9, 2491 10.1038/s41467-018-04895-1 29950568 PMC6021392

[79] Johnson A.A. and Johnson K.A. (2001) Exonuclease proofreading by human mitochondrial DNA polymerase. J. Biol. Chem. 276, 38097–38107 10.1074/jbc.M106046200 11477094

[80] Lee H.R. and Johnson K.A. (2006) Fidelity of the human mitochondrial DNA polymerase. J. Biol. Chem. 281, 36236–36240 10.1074/jbc.M607964200 17005554

[81] Plaza-G.A I., Lemishko K.M., Crespo R., Truong T.Q., Kaguni L.S., Cao-García F.J. et al. (2023) Mechanism of strand displacement DNA synthesis by the coordinated activities of human mitochondrial DNA polymerase and SSB. Nucleic Acids Res. 51, 1750–1765 10.1093/nar/gkad037 36744436 PMC9976888

[82] Korhonen J.A., Pham X.H., Pellegrini M. and Falkenberg M. (2004) Reconstitution of a minimal mtDNA replisome *in vitro*. EMBO J. 23, 2423–2429 10.1038/sj.emboj.7600257 15167897 PMC423294

[83] Sommers J.A., Estep K.N., Maul R.W. and Brosh R.M. Jr (2022) Biochemical analysis of DNA synthesis blockage by G-quadruplex structure and bypass facilitated by a G4-resolving helicase. Methods 204, 207–214 10.1016/j.ymeth.2021.12.005 34929333 PMC9203602

[84] Wanrooij P.H., Uhler J.P., Shi Y., Westerlund F., Falkenberg M. and Gustafsson C.M. (2012) A hybrid G-quadruplex structure formed between RNA and DNA explains the extraordinary stability of the mitochondrial R-loop. Nucleic Acids Res. 40, 10334–10344 10.1093/nar/gks802 22965135 PMC3488243

[85] Buchel G., Nayak A.R., Herbine K., Sarfallah A., Sokolova V.O., Zamudio-Ochoa A. et al. (2023) Structural basis for DNA proofreading. Nat. Commun. 14, 8501 10.1038/s41467-023-44198-8 38151585 PMC10752894

[86] Fernandez-Leiro R., Conrad J., Scheres S.H. and Lamers M.H. (2015) Cryo-EM structures of the *E. coli* replicative DNA polymerase reveal its dynamic interactions with the DNA sliding clamp, exonuclease and τ. Elife 4, 26499492 10.7554/eLife.11134 PMC4703070

[87] Fernandez-Leiro R., Conrad J., Yang J.-C., Freund S.M.V., Scheres S.H.W. and Lamers M.H. (2017) Self-correcting mismatches during high-fidelity DNA replication. Nat. Struct. Mol. Biol. 24, 140–143 10.1038/nsmb.3348 28067916 PMC5300136

[88] Roske J.J. and Yeeles J.T.P. (2024) Structural basis for processive daughter-strand synthesis and proofreading by the human leading-strand DNA polymerase Pol ε. Nat. Struct. Mol. Biol. 31, 1921–1931 10.1038/s41594-024-01370-y 39112807 PMC11638069

[89] Wang F., He Q., O'Donnell M.E. and Li H. (2025) The proofreading mechanism of the human leading-strand DNA polymerase ε holoenzyme. Proc. Natl. Acad. Sci. U.S.A. 122, e2507232122 10.1073/pnas.2507232122 40440070 PMC12146725

[90] Farge G., Pham X.H., Holmlund T., Khorostov I. and Falkenberg M. (2007) The accessory subunit B of DNA polymerase gamma is required for mitochondrial replisome function. Nucleic Acids Res. 35, 902–911 10.1093/nar/gkl1116 17251196 PMC1807957

[91] Copeland W.C. and Longley M.J. (2014) Mitochondrial genome maintenance in health and disease. DNA Repair (Amst.) 19, 190–198 10.1016/j.dnarep.2014.03.010 24780559 PMC4075028

[92] Sen D., Nandakumar D., Tang G.-Q. and Patel S.S. (2012) Human mitochondrial DNA helicase TWINKLE is both an unwinding and annealing helicase. J. Biol. Chem. 287, 14545–14556 10.1074/jbc.M111.309468 22383523 PMC3340288

[93] Hensen F., Potter A., van Esveld S.L., Tarrés-Solé A., Chakraborty A., Solà M. et al. (2019) Mitochondrial RNA granules are critically dependent on mtDNA replication factors Twinkle and mtSSB. Nucleic Acids Res. 47, 3680–3698 10.1093/nar/gkz047 30715486 PMC6468249

[94] Spelbrink J.N., Li F.Y., Tiranti V., Nikali K., Yuan Q.P., Tariq M. et al. (2001) Human mitochondrial DNA deletions associated with mutations in the gene encoding Twinkle, a phage T7 gene 4-like protein localized in mitochondria. Nat. Genet. 28, 223–231 10.1038/90058 11431692

[95] Tyynismaa H., Mjosund K.P., Wanrooij S., Lappalainen I., Ylikallio E., Jalanko A. et al. (2005) Mutant mitochondrial helicase Twinkle causes multiple mtDNA deletions and a late-onset mitochondrial disease in mice. Proc. Natl. Acad. Sci. U.S.A. 102, 17687–17692 10.1073/pnas.0505551102 16301523 PMC1308896

[96] Johnson L.C., Singh A. and Patel S.S. (2022) The N-terminal domain of human mitochondrial helicase Twinkle has DNA-binding activity crucial for supporting processive DNA synthesis by Polymerase γ. J. Biol. Chem. 299, 102797 36528058 10.1016/j.jbc.2022.102797 PMC9860392

[97] Ziebarth T.D., Gonzalez-Soltero R., Makowska-Grzyska M.M., Núñez-Ramírez R., Carazo J.-M. and Kaguni L.S. (2010) Dynamic effects of cofactors and DNA on the oligomeric state of human mitochondrial DNA helicase. J. Biol. Chem. 285, 14639–14647 10.1074/jbc.M109.099663 20212038 PMC2863210

[98] Farge G., Holmlund T., Khvorostova J., Rofougaran R., Hofer A. and Falkenberg M. (2008) The N-terminal domain of TWINKLE contributes to single-stranded DNA binding and DNA helicase activities. Nucleic Acids Res. 36, 393–403 10.1093/nar/gkm1025 18039713 PMC2241861

[99] Matsushima Y., Farr C.L., Fan L. and Kaguni L.S. (2008) Physiological and biochemical defects in carboxyl-terminal mutants of mitochondrial DNA helicase. J. Biol. Chem. 283, 23964–23971 10.1074/jbc.M803674200 18593709 PMC2527111

[100] Riccio A.A., Bouvette J., Perera L., Longley M.J., Krahn J.M., Williams J.G. et al. (2022) Structural insight and characterization of human Twinkle helicase in mitochondrial disease. Proc. Natl. Acad. Sci. U.S.A. 119, e2207459119 10.1073/pnas.2207459119 35914129 PMC9371709

[101] Ziebarth T.D., Farr C.L. and Kaguni L.S. (2007) Modular architecture of the hexameric human mitochondrial DNA helicase. J. Mol. Biol. 367, 1382–1391 10.1016/j.jmb.2007.01.079 17324440 PMC2711006

[102] Fernández-Millán P., Lázaro M., Cansız-Arda Ş., Gerhold J.M., Rajala N., Schmitz C.-A. et al. (2015) The hexameric structure of the human mitochondrial replicative helicase Twinkle. Nucleic Acids Res. 43, 4284–4295 10.1093/nar/gkv189 25824949 PMC4417153

[103] Peter B., Farge G., Pardo-Hernandez C., Tångefjord S. and Falkenberg M. (2019) Structural basis for adPEO-causing mutations in the mitochondrial TWINKLE helicase. Hum. Mol. Genet. 28, 1090–1099 10.1093/hmg/ddy415 30496414 PMC6423418

[104] Kaur P., Longley M.J., Pan H., Wang W., Countryman P., Wang H. et al. (2020) Single-molecule level structural dynamics of DNA unwinding by human mitochondrial Twinkle helicase. J. Biol. Chem. 295, 5564–5576 10.1074/jbc.RA120.012795 32213598 PMC7186178

[105] Wang H.-Y., Lee Y.-Y., Chien P.-J., Tsai W.-A., Sun P.-J., Wang L.-T. et al. (2025) Single-molecule fluorescence reveals the DNA unwinding mechanism of mitochondrial helicase TWINKLE and its interplay with single-stranded DNA-binding proteins. Nucleic Acids Res. 53, gkaf803 10.1093/nar/gkaf803 40867054 PMC12390753

[106] Johnson D.S., Bai L., Smith B.Y., Patel S.S. and Wang M.D. (2007) Single-molecule studies reveal dynamics of DNA unwinding by the ring-shaped T7 helicase. Cell 129, 1299–1309 10.1016/j.cell.2007.04.038 17604719 PMC2699903

[107] Ribeck N., Kaplan D.L., Bruck I. and Saleh O.A. (2010) DnaB helicase activity is modulated by DNA geometry and force. Biophys. J. 99, 2170–2179 10.1016/j.bpj.2010.07.039 20923651 PMC3042577

[108] Khan I., Crouch J.D., Bharti S.K., Sommers J.A., Carney S.M., Yakubovskaya E. et al. (2016) Biochemical characterization of the human mitochondrial replicative Twinkle helicase. J. Biol. Chem. 291, 14324–14339 10.1074/jbc.M115.712026 27226550 PMC4933186

[109] Enemark E.J. and Joshua-Tor L. (2006) Mechanism of DNA translocation in a replicative hexameric helicase. Nature 442, 270–275 10.1038/nature04943 16855583

[110] Thomsen N.D. and Berger J.M. (2009) Running in reverse: the structural basis for translocation polarity in hexameric helicases. Cell 139, 523–534 10.1016/j.cell.2009.08.043 19879839 PMC2772833

[111] Singh A., Patel G. and Patel S.S. (2023) Twinkle-catalyzed toehold-mediated DNA strand displacement reaction. J. Am. Chem. Soc. 145, 24522–24534 10.1021/jacs.3c04970 37917930 PMC11063129

[112] Doda J.N., Wright C.T. and Clayton D.A. (1981) Elongation of displacement-loop strands in human and mouse mitochondrial DNA is arrested near specific template sequences. Proc. Natl. Acad. Sci. U.S.A. 78, 6116–6120 10.1073/pnas.78.10.6116 6273850 PMC348988

[113] Madsen C.S., Ghivizzani S.C. and Hauswirth W.W. (1993) Protein binding to a single termination-associated sequence in the mitochondrial DNA D-loop region. Mol. Cell. Biol. 13, 2162–2171 8455604 10.1128/mcb.13.4.2162-2171.1993 PMC359537

[114] Roberti M., Musicco C., Polosa P.L., Milella F., Gadaleta M.N. and Cantatore P. (1998) Multiple protein-binding sites in the TAS-region of human and rat mitochondrial DNA. Biochem. Biophys. Res. Commun. 243, 36–40 10.1006/bbrc.1997.8052 9473475

